# Pseudomonas *palmensis* sp. nov., a Novel Bacterium Isolated From *Nicotiana glauca* Microbiome: Draft Genome Analysis and Biological Potential for Agriculture

**DOI:** 10.3389/fmicb.2021.672751

**Published:** 2021-08-20

**Authors:** Enrique Gutierrez-Albanchez, Ana García-Villaraco, José A. Lucas, Ignacio Horche, Beatriz Ramos-Solano, F. J. Gutierrez-Mañero

**Affiliations:** ^1^Biobab R&D S. L., Madrid, Spain; ^2^Plant Physiology, Pharmaceutical and Health Sciences Department, Faculty of Pharmacy, Universidad San Pablo-CEU Universities, Boadilla del Monte, Spain

**Keywords:** *Pseudomonas palmensis*, rhizobacteria, *Nicotiana glauca*, PGPB, iron mobilization, plant adaptation, drought, biostimulant

## Abstract

A novel *Pseudomonas*, designated strain BBB001^T^, an aerobic, rod-shaped bacterium, was isolated from the rhizosphere of *Nicotiana glauca* in Las Palmas Gran Canaria, Spain. Genomic analysis revealed that it could not be assigned to any known species of *Pseudomonas*, so the name *Pseudomonas palmensis* sp. nov. was proposed. A 16S rRNA gene phylogenetic analysis suggested affiliation to the *Pseudomonas brassicae* group, being *P. brassicae* MAFF212427 ^T^ the closest related type strain. Upon genomic comparisons of both strains, all values were below thresholds established for differentiation: average nucleotide identity (ANI, 88.29%), average amino acid identity (AAI, 84.53%), digital DNA-DNA hybridization (dDDH, 35.4%), and TETRA values (0.98). When comparing complete genomes, a total of 96 genes present exclusively in BBB001^T^ were identified, 80 of which appear associated with specific subsystems. Phenotypic analysis has shown its ability to assimilate glucose, potassium gluconate, capric acid malate, trisodium citrate, and phenylacetic acid; it was oxidase positive. It is able to produce auxins and siderophores *in vitro*; its metabolic profile based on BIOLOG Eco has shown a high catabolic capacity. The major fatty acids accounting for 81.17% of the total fatty acids were as follows: C_16:0_ (33.29%), summed feature 3 (22.80%) comprising C_16:1_ ω7*c* and C_16:1_ ω6*c*, summed feature 8 (13.66%) comprising C_18:1_ ω7*c*, and C_18:1_ω6*c* and C_17:0_ cyclo (11.42%). The ability of this strain to improve plant fitness was tested on tomato and olive trees, demonstrating a great potential for agriculture as it is able to trigger herbaceous and woody species. First, it was able to improve iron nutrition and growth on iron-starved tomatoes, demonstrating its nutrient mobilization capacity; this effect is related to its unique genes related to iron metabolism. Second, it increased olive and oil yield up to 30% on intensive olive orchards under water-limiting conditions, demonstrating its capacity to improve adaptation to adverse conditions. Results from genomic analysis together with differences in phenotypic features and chemotaxonomic analysis support the proposal of strain BBB001^T^ (=LMG 31775^T^ = NCTC 14418^T^) as the type strain of a novel species for which the name *P. palmensis* sp. nov is proposed.

## Introduction

Plant growth–promoting bacteria (PGPBs) are bacteria that colonize plant roots and promote plant growth. These bacteria live in the rhizosphere and play a very important role in plant fitness; in some species, they live in symbiosis with the plant, whereas in most of them, they establish a loose relationship colonizing the root surface. Plants often depend on these PGPBs to acquire nutrients, whereas in other cases, PGPB provides protection against certain pathogens, produces phytohormones such as auxins improving plant development, or helps the plant to get adapted to biotic and abiotic stresses such as herbivores, pathogenic microorganisms, or salinity (Barriuso et al., 2005; Delfin et al., 2015; Bano and Muqarab, 2017).

*Nicotiana glauca* is a plant species from the *Solanaceae* family, native from South America and naturalized in the Mediterranean area and in the Canary Islands. Able to colonize poor soils, *N. glauca* contains anabasine, an alkaloid that confers some toxicity, and indicates the existence of a complex secondary metabolism, inducible and probably related to defense and plant adaptation (Sinclair et al., 2004; DeBoer et al., 2009). Therefore, wild populations of *N. glauca* colonizing harsh environments will select the best candidates for adaptation and would be a good source for putative PGPBs (Ramos-Solano et al., 2010). Many different bacterial genera have been reported to be PGPBs, and among them, *Pseudomonas* strains are very abundant. They are Gram-negative flagellated bacteria, often known because of the pigment production; they play crucial roles in soil health and plant development (Kloepper, 1992) and affect plant growth (Van Loon, 2007). Among the mechanisms frequently used by *Pseudomonas* species to benefit plant growth are siderophore production (Mavrodi et al., 2001; Rasouli-Sadaghiani et al., 2014), phosphate solubilization (Anzuay et al., 2013), or stimulation of plant protection triggering induced systemic resistance (Bakker et al., 2007; Fatima and Anjum, 2017).

As part of an ongoing research, bacteria from the rhizosphere of *N. glauca* were isolated, and strain BBB001^T^ was characterized. In this study, a full genetic analysis was conducted to investigate the phylogenetic relationship of strain BBB001^T^ within the *Pseudomonas* genus by sequencing the whole genome and comparing with those of the most similar genomes within this genus. A polyphasic characterization was also carried out. In addition, two biological assays were performed to support its potential for agriculture, one to demonstrate its ability to improve iron nutrition in tomato and another one to increase olive production under water-limiting conditions.

## Results

### Phenotypic Characterization

Strain BBB001^T^ isolated from the rhizosphere of *N. glauca* was classed as a Gram-negative, non–endospore-forming rod. Strain BBB001^T^ grows on plate count agar (PCA) at 28°C forming circular colonies < 1 mm ∅, with smooth borders, opaque, yellowish, and creamy texture. In liquid culture (nutrient broth), color changes from pale yellow in the exponential growth phase to intense yellow on the stationary phase after 24 h at 28°C.

Temperature growth test shows that the strain BBB001^T^ grows in 24 h from 4 to 40°C, although at low temperatures (4°C) it takes up to 72 h. As regards pH, strain BBB001^T^ grows in a pH range from 6 to 10 and with a salt concentration from 2 to 6%. The optimal temperature, pH, and salinity of growth for strain BBB001^T^ are as follows: 28°C, pH 8, and 0% salinity.

The API20 NE reveals its ability to assimilate several substrates, namely, glucose, potassium gluconate, capric acid, malate, trisodium citrate, and phenylacetic acid; it is oxidase positive (Table 1). Compared to *Pseudomonas brassicae* MAFF212427^T^ and *Pseudomonas laurentiana* GSL-010^T^, strain BBB001^T^ is able to degrade CAP, whereas *P. brassicae*^T^ cannot, and it is oxidase positive, whereas *P. laurentiana*^T^ is not, sharing all other metabolic capabilities with the two type strains.

**TABLE 1:** Characteristics that differentiate *Pseudomonas palmensis* sp. nov. from the type strains of the most closely related *Pseudomonas* species: *Pseudomonas brassicae* MAFF212427 ^T^ (Sawada et al., 2020) and *Pseudomonas laurentiana* GSL-010 ^T^ (Wright et al., 2018).

	Strain BBB001^T^	*Pseudomonas brassicae* MAFF 212427^T^	*Pseudomonas laurentiana* GSL-010^T^
Cell size (μm)	0.5−0.8 × 1.5−3.8	2.1 ± 0.4 × 1.2 ± 0.1	1.75−2.2 × 0.5−0.7
Isolation source	Rhizosphere (Spain)	Diseased brocoli	Seawater (Canada)
Temperature range (optimal) (°C)	4–40 (28)	4–35 (27)	10–37 (30)
NaCl optimal (%, w/v)	0	ND	0.5
Maximum NaCl (%, w/v)	6	ND	3
pH range	6–10	ND	5–10
pH optimum	8	ND	7–7.5
**API20 NE**			
[CAP]	+	−	+
OX	+	+	−
**Saturated fatty acid**			
C_10:0_	−	−	0.11
C_12:0_	4.02	4.00	2.66
C_14:0_	0.94	−	1.76
C_15:0_	−	_	0.42
C_16:0_	33.29	28.10	32.29
C_17:0_	−	_	0.13
C_18:0_	0.52	_	0.18
**Unsaturated fatty acid**			
C_16:1_ ω 5*c*	−	_	0.11
C_17:1_, ω 8*c*	−	_	0.10
C_18:1_, ω 7*c*	−	_	8.30
**Branched fatty acid**			
C_17:0_ cyclo	11.42	7.80	1.13
C_18:1_ 11 methyl ω7C	−	−	0.35
C_19:0_ cyclo ω8*c*	0.81	−	−
**Hydroxy fatty acid**			
C_8:0_ 3-OH	−	−	−
C_10:0_ 3-OH	9.96	4.00	3.83
C_11:0_ 3-OH	−	−	−
C_12:0_ 2-OH	4.23	2.60	4.18
C_12:0_ 3-OH	4.35	3.90	3.66
C_12:1_ 3-OH	−	−	0.07
Summed Features*			
2	−	−	−
3	22.80	29.00	40.43
7	−	−	0.17
8	13.66	17.5	0
**Utilization of:**			
L-Phenylalanine	+	Unkown	−

For API20 NE results obtained in this study, all three strains were negative for NO3, TRP, GLU, ADH, URE, ESC, GEL, PNPG, GLU, ARA, MNE, MAN, NAG, MAL, GNT, and ADI and positive for GNT, GLU, MLT, CIT, and PAC. Characteristics for API20 NE and biolog plates are scored as follows: − negative reaction; + positive reaction. Fatty acid profile expressed as% (FAME): − indicates absence of the fatty acid. Summed feature* represents two or three fatty acids that cannot be separated using the MIDI system. Summed feature 3 comprises C_16:1_ ω7c/C_16:1_ ω 6c; summed feature 8 comprises C_18:1_ ω 7c/C_18:1_ ω 6c.

According to BIOLOG ECO, strain BBB001^T^ is able to degrade several carboxylic acids, among which it is worth noting malic acid, hydroxybutyric acid and glucosaminic acid, the latter can also be consumed as a source of nitrogen. Among sugars, glucose-1-phosphate, cellobiose, lactose, and *N*-acetyl glucosamine stand out. As a nitrogen source, strain BBB001^T^ catabolizes the two amines (putrescine and phenyl ethylamine), although the consumption of some amino acids, such as serine and glycol-L-glutamic acid, is also remarkable (Supplementary Table 2).

The major fatty acids (>81.17% of the total fatty acids) are C_16:0_ (33.29%), summed feature 3 (22.80%) comprising C_16:1_ ω7*c* and C_16:1_ ω6*c*, summed feature 8 (13.66%) comprising C_18:1_ ω7*c*, and C_18:1_ ω6*c* and C_17:0_ cyclo (11.42%).

In addition, this strain resulted positive for auxins as for siderophores synthesis and negative for chitinases production.

In addition to API20 information, Table 1 summarizes available information from the most closely related *Pseudomonas* species, confirming differences for strain BBB001^T^. Interestingly, and different from BBB001^T^, “*Pseudomonas qingdaonensis*” strain JJ3^T^ was not able to degrade D-galacturonic acid, and its temperature range runs from 4 to 37°C, whereas BBB001^T^ runs from 4 to 40°C (Sawada et al., 2020).

### Phylogenetic Analysis

The genome of the BBB001^T^ strain was analyzed with the tools available at EzBioCloud. First, EzBioCloud’s identification service provides proven similarity-based searches against quality-controlled databases of 16S rRNA sequences. After that, the ANI (average nucleotide identity), AAI (average amino acid identity), dDDH, and TETRA analysis were performed. Type (Strain) Genome Server (TYGS) was used to analyze the complete genome.

#### Estimates of the Degree of Similarity Between Genomes

**FIGURE 1:**
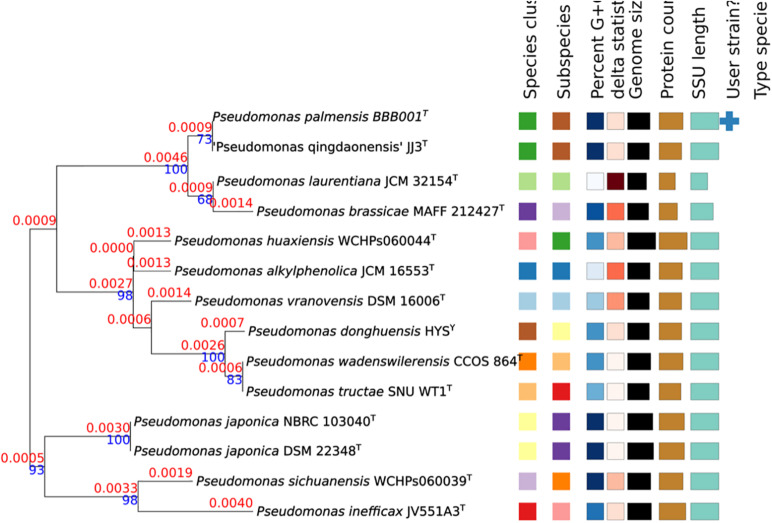
Tree inferred with FastME 2.1.6.1 (Lefort et al., 2015) from GBDP distances calculated from 16s rRNA gene sequences. The branch lengths are scaled in terms of GBDP distance formula d5. The blue numbers are GBDP pseudo-bootstrap support values > 60% from 100 replications, with an average branch support of 80.9%. The branch length values (in red) represent the evolutionary time between two nodes. Unit: substitutions per sequence site. The tree was rooted at the midpoint (Farris, 1972).

(a) Phylogenetic analysis and delimitation of species based on the analysis of 16S. The size of 16S rRNA gene was 1,541 bp (MW009702). The analysis based on 16S rRNA gene indicated that the strain BBB001^T^ shared the highest similarity with *P. qingdaonensis* JJ3^T^ (100%, non-validated taxon), *P. brassicae* MAFF212427^T^ (99.85%), and *Pseudomonas defluvii* WCHP16^T^ (99.31%), whereas *P. laurentiana* GSL-010^T^, *Pseudomonas japonica* NBRC 103040^T^, and *Pseudomonas huaxiensis* WCHPS060044^T^ were between 99.00 and 99.1% (Table 2). A phylogenetic tree was constructed based on comparison of the most similar 16S rRNA gene sequences available in the TYGS database (Figure 1). Three different groups appeared in the tree: group 1 in which *Pseudomonas japonica* NBRC 103040^T^, *Pseudomonas sinwaenesis* WCHPs060039^T^, and *Pseudomonas ineficax* JV551A3^T^ were located; group 2, represented by *Pseudomonas alkylphenolica* KL28^T^, *Pseudomonas huaxinesis* WCHPs060044^T^, *Pseudomonas vranoviensis* CCM 7279^T^, *Pseudomonas donghuensis* HYS^T^, *Pseudomonas wandersilveriensis* CCOS 864^T^, and *Pseudomonas tructae* SNU WT1^T^; finally group 3, involving *P. qingdaonensis* JJ3(T), strain BBB001, *P. brassicae* MAFF212427^T^, *P. laurentiana* GSL-010^T^, and *Pseudomonas akapagensis* PS24^T^. In the third group, *P. laurentiana* GSL-010^T^ and *P. akapagensis* PS24^T^ separated from the other three, showing that *P. qingdaonensis* JJ3(T) and strain BBB001 grouped together, separate from *P. brassicae* MAFF212427^T^, which is the closest phylogenetically related strain.

**TABLE 2:** Similarity of the 16S rRNA of the 30 taxa with valid names and strain BBB001^T^.

Rank	Name	Type Strain (T)	Accession	Pairwise Similarity(%)	Authors
1	Pseudomonas qingdaonensis’	JJ3	PHTD01000020	100.0	Wang et al., 2019
2	*Pseudomonas brassicae*	MAFF 212427	LC514379	99.9	Sawada et al., 2020
3	*Pseudomonas defluvii*	WCHP16	KY979145	99.3	Qin et al., 2020
4	*Pseudomonas laurentiana*	GSL-010	KY471137	99.1	Wright et al., 2018
5	*Pseudomonas japonica*	NBRC 103040	BBIR01000146	99.0	Pungrasmi et al., 2008
6	*Pseudomonas huaxiensis*	WCHPs060044	MH428812	99.0	Qin et al., 2019a
7	*Pseudomonas alkylphenolica*	KL28	CP009048	98.9	Mulet et al., 2015
8	*Pseudomonas graminis*	DSM 11363	Y11150	98.8	Behrendt et al., 1999
9	*Pseudomonas donghuensis*	HYS	AJJP01000212	98.8	Gao et al., 2015
10	*Pseudomonas sichuanensis*	WCHPs060039	QKVM01000121	98.8	Qin et al., 2019b
11	*Pseudomonas rhizosphaerae*	DSM 16299	CP009533	98.6	Peix et al., 2003
12	*Pseudomonas wadenswilerensi*	CCOS 864	LT009706	98.6	Frasson et al., 2017
13	*Pseudomonas plecoglossicida*	NBRC 103162	BBIV01000080	98.6	Nishimori et al., 2000
14	*Pseudomonas wanovensis*	CCM 7279	AY970951	98.6	Tvrzova et al., 2006
15	*Pseudomonas asiatica*	RYU5	MH517510	98.6	Tohya et al., 2019a
16	*Pseudomonas taiwanensis*	BCRC 17751	EU103629	98.6	Wang et al., 2010
17	*Pseudomonas putida*	NBRC 14164	AP013070	98.5	Trevisan, 1889; Migula, 1895
18	*Pseudomonas inefficax*	JV551A3	OPYN01000008	98.5	Keshavarz-Tohid et al., 2019
19	*Pseudomonas asplenni*	ATCC 23835	LT629777	98.4	Ark and Tompkins, 1946; Savulescu, 1947
20	*Pseudomonas monteilii*	NBRC 103158	BBIS01000088	98.4	Elomari et al., 1997
21	*Pseudomonas tructae*	SNU WT1	CP035952	98.4	Oh et al., 2019
22	*Pseudomonas coleopterorum*	Esc2Am	KM888184	98.4	Menéndez et al., 2015
23	*Pseudomonas reidholzensis*	CCOS 865	LT009707	98.4	Frasson et al., 2017
24	*Pseudomonas juntendi*	BML3	MK680061	98.4	Tohya et al., 2019b
25	*Pseudomonas cremoricolorata*	1AM 1541	AB060137	98.3	Uchino et al., 2001
26	*Pseudomonas entomophila*	L48	CT573326	98.3	Mulet et al., 2012
27	*Pseudomonas mosselii*	CIP 105259	AF072688	98.3	Dabboussi et al., 2002
28	*Pseudomonas moorei*	RW10	AM293566	98.3	Cámara et al., 2007
29	*Pseudomonas lutea*	DSM 17257	JRMB01000004	98.2	Peix et al., 2004
30	*Pseudomonas helmanticensis*	OHA11	HG940537	98.2	Ramírez-Bahena et al., 2014
31	*Pseudomonas baetica*	a390	FM201274	98.2	López et al., 2012

*In column 1, accession number is indicated after the species name.*

(b) The highest values of ANI and AAI (88.29 and 84.83%, respectively) were obtained with the genome of the species *P. brassicae* MAFF212427^T^. The highest value of dDDH (35.4%) and the lower intergenomic distance (0.11) was obtained when comparing strain BBB001^T^ and *P. brassicae* MAFF212427^T^ (Table 1). G + C content shows a difference of 0.65% between genomes of these two strains. TETRA values were between 0.86 and 0.988, not showing a significant resemblance between the genome of strain BBB001^T^ and the other 24 genomes (Table 3). Comparison with the genome of *P. qingdaonensis* JJ3(T) (non-validated taxon) showed the highest values (Table 3).

**TABLE 3:** Similarity of the 24 genomes with strain BBB001^T^ genome.

Rank	Name	genome accesion number	ANI%	AAI%	dDDH	Intergenomic distance	Difference G + C (%)	TETRA
1	Pseudomonas qingdaonensis’ JJ3(T)	PHTD00000000.1	99.2	95.0	93.4	0.0855	0.14	0.9998
2	*Pseudomonas brassicae* MAFF 212427(T)	LC514391.1	88.3	84.5	35.4	0.1167	0.65	0.9880
3	*Pseudomonas laurentiana* JCM 32154(T)	JAAHBT000000000.1	81.6	81.0	25.1	0.1732	4.35	0.8680
4	*Pseudomonas japonica* NBRC 103040(T)	NZ BBIR00000000.1	83.6	78.0	26.7	0.1618	0.10	0.9600
5	*Pseudomonas alkylphenolica* KL28(T)	NZ CP009048.1	83.4	80.0	27.1	0.1597	3.43	0.9384
6	*Pseudomonas graminis* DSM 11363(T)	NZ FOHW00000000.1	77.9	72.5	22.5	0.1951	3.8	0.9040
7	*Pseudomonas donghuensis* HYS(T)	NZ AJJP00000000.1	84.2	78.0	27.7	0.1554	1.64	0.9699
8	*Pseudomonas sichuanensis* WCHPs060039(T)	NZ QKVM00000000.1	82.0	76.8	25.3	0.1716	0.29	0.9840
9	*Pseudomonas rhizosphaerae* DSM 16299 (T)	CP009533.1	79.7	72.6	23.5	0.1859	2.06	0.9550
10	*Pseudomonas wadenswilerensis* CCOS 864(T)	NZ UIDD00000000.1	84.1	78.8	27.7	0.1556	1.67	0.9680
11	*Pseudomonas plecoglossicida* NBRC 103162(T)	NZ BBIV00000000.1	81.9	74.9	25.1	0.1732	1.26	0.9820
12	*Pseudomonas vranovensis* CCM 7279(T)	NZ AUED00000000.1	83.3	81.1	26.6	0.1630	2.53	0.9610
13	*Pseudomonas asiatica* RYU5(T)	NZ BUF01000001.1	81.9	77.0	25.3	0.1718	1.24	0.9760
14	*Pseudomonas taiwanensis* BCRC 17751(T)	NZ AUEC00000000.1	81.0	77.9	24,00	0.1823	2.19	0.9610
15	*Pseudomonas putida* NBRC 14164(T)	NC 021505.1	81.4	76.3	25,00	0.1745	1.72	0.9700
16	*Pseudomonas inefficax* JV551A3(T)	OPYN00000000.1	81.8	75.8	25.5	0.1706	1.21	0.9718
17	*Pseudomonas monteilii* NBRC 103158(T)	NZ BBIS00000000.1	81.5	77.1	24.7	0.1768	2.37	0.9670
18	*Pseudomonas coleopterorum* LMG 28558(T)	FNTZ00000000.1	79.6	74.3	23.4	0.1866	2.17	0.9542
19	*Pseudomonas reidholzensis* CCOS 865(T)	UNOZ00000000	82.1	76.2	25,00	0.1741	0.03	0.9700
20	*Pseudomonas cremoricolorata* 1AM 1541(T)	NZ AUEA00000000.1	80.7	73.9	23.6	0.1856	0.55	0.9531
21	*Pseudomonas mosselii* CIP 105259(T)	NZ MRVJ00000000.1	82.3	77.4	25.4	0.1715	0.38	0.9810
22	*Pseudomonas moorei* DSM 12647(T)	NZ FNKJ01000003.1	79.4	72.8	23.6	0.1850	4.40	0.9120
23	*Pseudomonas lutea* DSM 17257(T)	JRMB00000000.1	77.4	74.4	22.5	0.1946	3.91	0.8920
24	*Pseudomonas baetica* a390(T)	PKLC00000000.1	78.8	73.4	22.9	0.1916	5.30	0.8681

*For each genome, its length [in base pairs (bp)], number of annotated genes, and the values of ANI, AAI, dDDH, intergenomic distance, G + C difference, and TETRA index estimated in the comparison with the genome of Pseudomonas BBB001^T^. In column 1, the genome accession number is indicated after the species name in RefSeq.*

#### Phylogenetic Analyses Based on Complete Genome Sequences

**FIGURE 2:**
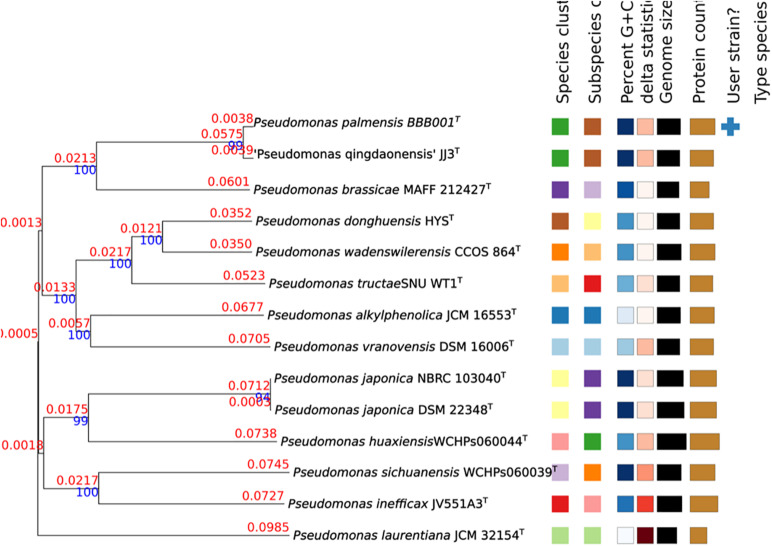
Phylogenetic tree constructed in TYGS. Tree inferred with FastME 2.1.6.1 (Lefort et al., 2015) from GBDP distances calculated from genome sequences. The branch lengths are scaled in terms of GBDP distance formula d5. The numbers above the branches are GBDP pseudo-bootstrap support values > 60% from 100 replications, with an average branch support of 94.3%. The branch length values represent the evolutionary time between two nodes. Unit: substitutions per sequence site. The tree was rooted at the midpoint (Farris, 1972).

The genome of strain BBB001^T^ ^1^ (WGS: JAFKEC000000000) was compared against all type strain genomes available in the TYGS database *via* the MASH algorithm, a fast approximation of intergenomic relatedness (Ondov et al., 2016), and the 10 type strains with the smallest MASH distances chosen per user genome resulting in a phylogenetic tree (Figure 2).

**FIGURE 3:**
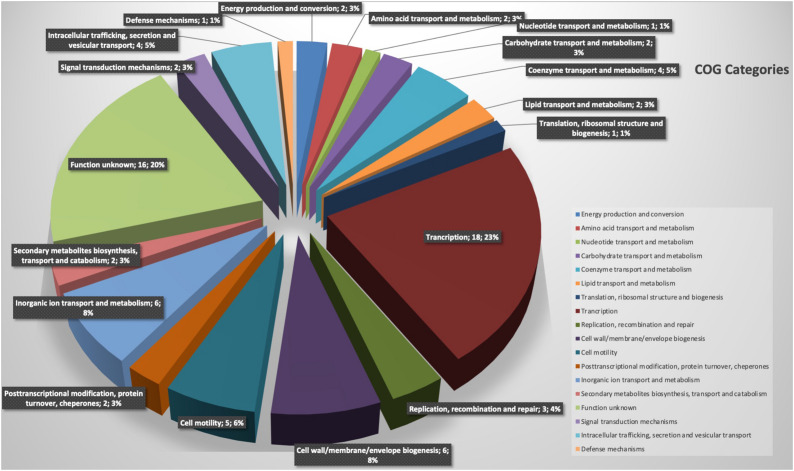
Representation of functional categories of novel regions of *strain* BBB001^T^.

The comparative analysis of the complete genomes identified a total of 79 genes present exclusively in strain BBB001^T^ (Supplementary Table 2). Among these genes (Figure 3), transcription-related genes were the most abundant (18) representing 23% or these unique genes; 16 genes were not associated with specific subsystems (20%); 6 were associated with cell wall, membrane, and envelope synthesis (8%); and 6 (8%) were related to inorganic transport and metabolism (eggnog-emapper). Further analysis was carried out on genes related to inorganic transport and metabolism, and two genes related to the uptake and iron metabolism were identified by blast in antiSMASH 5.0^2^ secondary metabolism database; this is consistent with data from phenotypic characterization.

### Biological Assays

**FIGURE 4:**
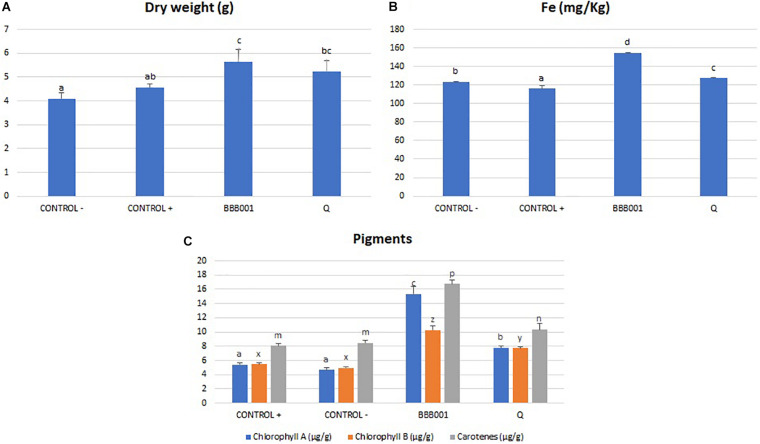
**(A)** Dry weight of tomato plants (g). **(B)** Iron plant content (mg/kg). **(C)** Pigment concentration (mg/g) on tomato leaves. Different letters denote statistically significant differences according to LSD test (*p* < 0.05) for chlorophyl a (a, b, and c), chlorophyl b (x, y, and z), and carotenes (m, n, and p).

The biological assay on iron-starved tomato was conducted to proof the biological effect associated with iron absorption (Fe^3+^) and metabolism unique genes of this strain (Figure 3). Data showed that both strain BBB001^T^ and its culture media free of bacteria (chelate) supplemented with non-soluble iron were able to revert chlorosis due to iron deficiency, according to visual evaluation (data not shown). Strain BBB001^T^ significantly increased dry weight (Figure 4A), Fe concentration (Figure 4B), and photosynthetic pigments (chlorophylls and carotenes, Figure 4C), against the positive control (Fe-EDTA) and the negative control. The chelate produced by the bacteria significantly increased all parameters, except the increase in dry weight, which was non-significant; increases were not as marked as with the bacteria (Figure 4).

**FIGURE 5:**
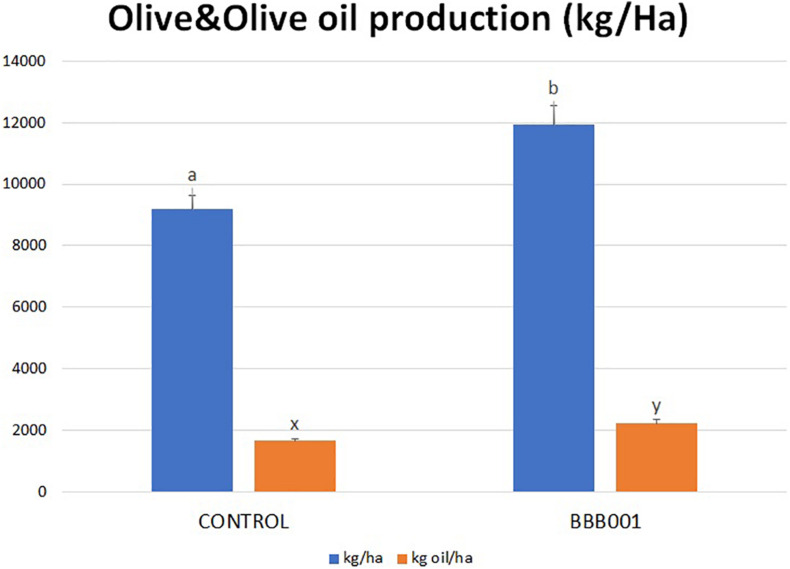
Olive and olive oil production of *Olea europaea* trees (kg/ha). Different letters denote statistically significant differences according to LSD test (*p* < 0.05) for olive yield (a, b), oil yield (x, y).

The biological assay in the intensive olive orchard was conducted aiming to demonstrate the ability of the strain to improve plant adaptation to stress situations, decreasing water input by 25% of regular watering. Results showed a statistically significant increase in olive yield up to 30% (kg/ha), as well as in oil production (Figure 5). Interestingly, the production achieved with strain BBB001^T^ under water-limiting conditions (12,000 kg/ha) reached similar values to controls under regular water regime (data not shown).

## Discussion

Strain BBB001^T^ isolated from the rhizosphere of *N. glauca* is able to improve plant yield in olive trees under water-limiting conditions and to efficiently supply iron to tomato under iron-limiting conditions, demonstrating its good potential for agriculture. Although the beneficial effects in plants have been described before for other *Pseudomonas* (Kloepper, 1992; Mavrodi et al., 2001; Bakker et al., 2007; Van Loon, 2007; Anzuay et al., 2013; Rasouli-Sadaghiani et al., 2014; Fatima and Anjum, 2017), the multiphasic genomic analysis has evidenced that this is a new strain with beneficial properties for agriculture, for which the name *Pseudomonas palmensis* sp. nov. has been proposed.

The ability of *N. glauca* to select the best strains to improve adaptation to harsh environments has been evidenced consistent with previous studies (Ramos-Solano et al., 2010). Its complex secondary metabolism, inducible and probably related to defense and plant adaptation (Sinclair et al., 2004; DeBoer et al., 2009), together with scarcity of nutrients, is key to select strains that play crucial roles in soil health and plant development. In line with this statement, strain BBB001^T^ was positive for auxins and for siderophore production. The ability to produce auxins is very common and is related to increases in root surface, which provide plants with an enhanced capacity to absorb water and nutrients and therefore increased growth potential (Gutierrez Mañero et al., 1996).

The carbon source utilization fingerprint characteristic of strain BBB001^T^ indicates a great adaptive capacity to survive in the rhizosphere of plants, quickly metabolizing organic acids and carbohydrates released by roots, which makes this strain a good root colonizer, one of the traits necessary for effective PGPBs (Posada et al., 2018). This is consistent with bioassay results, as field-grown olives evidence an increase in yield due to bacterial effects, probably due to an efficient colonization.

As there is an increasing number of PGPBs being described, many of which belong to *Pseudomonas*, a multiphasic approach to study its genome was taken to unravel the hidden information of this strain with such a good performance. First, analysis of the 16S rRNA revealed a high similarity (>99%) to five species with validated names. Sequencing of the 16S ribosomal gene (16S rRNA) is the basic tool in the current system classification of bacterial species. Generally, similarity values for the 16S rRNA gene less than 98.65 to 99% are accepted for different species (Kim et al., 2014). However, the 16S rRNA gene may offer limited resolution power as it shows a low capacity for discrimination between closely related species. Furthermore, some species may show very similar sequences of 16S rRNA (>99%), despite being clearly different according to DDH values (Ash et al., 1991; Rosselló-Móra and Amann, 2001). Consistently, ANI and AAI values confirmed differences to existing validated genomes. The values of ANI and AAI represent a robust measure of the evolutionary distance between genomes, as it has been empirically verified that ANI and AAI values of 95 to 96% equals a DDH value (DNA–DNA hybridization) of 70%, commonly used to delimit prokaryotic species (Konstantinidis and Tiedje, 2005; Richter and Rosselló-Móra, 2009; Kim et al., 2014). The closer related strain genomes will show similar frequencies of tetranucleotide use, with correlation indexes (TETRA) equal to or greater than 0.99 (Teeling et al., 2004; Richter and Rosselló-Móra, 2009). The highest values of ANI and AAI obtained in the comparison with the genome of the species *P. brassicae* were well below the 95% threshold established for considering that both genomes belong to the same species. The highest value of dDDH and the lower intergenomic distance were obtained when comparing strain BBB001^T^ and *P. brassicae* MAFF212427^T^ (Table 3), being the value of dDDH much lower than the 70% necessary to be able to assign strain BBB001^T^ to this species. This result is also corroborated by TETRA values, which did not show a significant resemblance between the genome of strain BBB001^T^ and the other genomes with values less than 0.99%. In the phylogenetic tree constructed in TYGS with complete genome data (Figure 2), strain BBB001^T^ is closely related with *P. brassicae* MAFF212427^T^ and *P. qingdaonensis* JJ3^T^. In this case, the analysis establishes a closer relationship between strain BBB001^T^ and *P. qingdaonensis* JJ3^T^, which taxonomic name is not validated yet^3^. It is likely therefore that the repertoire of accessory genes of strain BBB001^T^ is more like that of *P. qingdaonensis* JJ3^T^ than that of *P. brassicae* MAFF212427^T^.

Comparison of the most similar complete genomes at Pan-seq revealed novel regions of this strain, a total of 96 genes were present exclusively in strain BBB001^T^, and 80 of these genes appear associated with specific subsystems. Among these genes (Figure 3), those related to the uptake and iron metabolism were found (Coulton et al., 1983; Braun, 2003; Miethke and Marahiel, 2007) and correlate with data from phenotypic characterization.

Biological assays were designed to evidence the potential of the strain for agriculture based on its unique genes related to iron metabolism on the one hand and of its general performance. The biological assay on iron-starved tomato was conducted based on information about unique genes of this strain related to iron absorption (Fe^3+^) and metabolism (Figure 3). Data showed that both BBB001^T^ and its culture media free of bacteria (chelate) supplemented with non-soluble iron were able to revert chlorosis due to iron deficiency, according to visual evaluation, significantly increasing dry weight, iron concentration and photosynthetic pigments in iron-starved tomatoes, being the bacteria much more efficient than the chelate (Figure 4).

The differences between the bacteria and the culture media suggest that the increased iron mobilization is strongly improved when the bacteria are present, consistent with the multitarget response proposed for beneficial bacteria (Ilangumaran and Smith, 2017). In this type of response, bacteria improve iron mobilization probably supported by an increased root development that allows an increase in absorption surface (Gutierrez Mañero et al., 1996), a response attributed to auxin concentration, also present in the unique gene set of the strain. The beneficial effect on iron nutrition also shows an increase of photosynthetic pigments on the shoot, being especially striking the increase on chlorophyll a, the key player on light reactions as it is mainly located on photosystems. The increase in photosynthetic potential is evidenced on growth (dry weight), with the highest increase in dry weight on the bacteria treated plants, confirming that not only iron limits plant growth and supporting the better performance of bacteria in agriculture, where many environmental factors change along plant growth cycle.

The biological assay in the intensive olive orchard was conducted aiming to demonstrate the ability of the strain to improve plant adaptation to stress situations, especially the lack of water, as this situation becomes more and more frequent nowadays. The mechanisms involved in plant adaptation to stress are common to many other stressful situations such as salinity or biotic stress, through common signaling pathways (Martin-Rivilla et al., 2020). Plant adaptive mechanisms involve ionic homeostasis and modulation of redox balance among others (Kangasjärvi et al., 2014; Kollist et al., 2019). Olive yield (kg/ha) and oil production increased significantly (up to 30%) despite the 25% decrease of water input (Figure 4). The registered increase in oil was due to two factors: the increment in olive production and the increase in fat yield, reaching similar values to controls under regular water regimen (data not shown).

These results support the suitability of this strain to activate adaptive mechanisms in different plant species, from herbaceous to woody plant species, as well as its versatility to different environmental stress factors, evidencing its multitarget power (Ilangumaran and Smith, 2017).

Genomic analysis on strain BBB001 revealed that it could not be assigned to any known species of *Pseudomonas*, so the name *P. palmensis* sp. nov. was proposed for strain BBB001^T^. A 16S rRNA gene sequence–based phylogenetic analysis suggested that the strain BBB001^T^ was affiliated with the *P. brassicae* group with *P. brassicae* MAFF212427^T^ as the most closely related type strain (99.85% similarity). Genomic comparisons of strain BBB001^T^ with the type strain species *P. brassicae* MAFF212427^T^ were all below thresholds established for differentiation. In the comparative analysis of complete genomes, a total of 96 genes present exclusively in strain BBB001^T^ were identified, 80 of which appear associated with specific subsystems.

Also, the novel taxon possesses several phenotypic traits related to beneficial plant traits, like auxin production or iron mobilization. Results obtained in iron-starved tomato and water-limited olive orchards confirm the potential of this strain for agriculture. On the one hand, it is efficient for iron mobilization under nutrient stress and adverse conditions, consistent with the specific and unique set of genes associated with iron metabolism, so it can be used to develop organic Fe supplies for sustainable agriculture, both the strain and its metabolites. On the other hand, it can improve adaptation to water-limiting conditions while keeping high productivity, so it is an excellent candidate to develop biotechnological products for agriculture under harsh conditions. Finally, the effects recorded in herbaceous species such as tomato, as well as in a woody species such as olive, confirm its wide scope capability to succeed in most agronomic crops, as this strain can trigger plant metabolism in different species.

In summary, all phenotypic and genomic characterization of the novel strain BBB001^T^ reveals a distinct and well-differentiated species from all *Pseudomonas* species with validated reference taxons and, more precisely, different from *P. brassicae* MAFF212427^T^.

Thus, based on the polyphasic approach, we describe a novel *Pseudomonas* species, for which the name *P. palmensis* sp. nov. is proposed, based on the geographical origin of isolation, Las Palmas de Gran Canarias (Islas Canarias, Spain). The type strain is strain BBB001^T^ (= LMG 31775 = NCTC 14418).

### Description of *Pseudomonas palmensis* sp. nov

*Pseudomonas palmensis* (palm.en’sis), N.L. masc./fem. adj. palmensis, belonging to Las Palmas de Gran Canaria, Spain, from which the type strain was isolated.

Cells are Gram-reaction negative, aerobic, rod-shaped bacteria ranging between 0.5 and 0.8 μm × 1.5–3.8 μm long, non–endospore-forming rods. Colonies grow on PCA at 28°C forming circular colonies < 1 mm ∅, with smooth borders, opaque, yellowish, and creamy texture. In liquid culture (nutrient broth), color changes from pale yellow in the exponential growth phase to intense yellow on the stationary phase after 24 h at 28°C. Growth occurs at 4 to 40°C in 24 h but not at 42°C. As regards pH, strain BBB001^T^ grows in a pH range from 6 to 10 and with a salt concentration from 2 to 6%. The optimal temperature, pH, and salinity of growth for BBB001^T^ are 28°C, pH 8, and 0% salinity. It is able to assimilate glucose, potassium gluconate, capric acid malate, trisodium citrate, and phenylacetic acid; it is oxidase positive. Its metabolic profile based on BIOLOG Eco shows a high catabolic capacity. It is able to produce auxins and siderophores, but not chitinases. The major fatty acids (> 81.17% of the total fatty acids) are C_16:0_ > summed feature 3 (C_16:1_ω7*c*/C_16:1_ ω6*c*), > summed feature 8 (C_18:1_ω7*c*/C_18:1_ω6*c*) > C_17:0_ cyclo.

The strain BBB001^T^ was isolated form the rhizosphere of *N. glauca* L. in Las Palmas de Gran Canaria Spain, in 2017. The genomic DNA G + C content of the type strain is 64.05%. The draft genome and 16S rRNA gene sequences of the strain BBB001^T^ have been deposited at the NCBI GenBank under accession numbers MW009702 and JAFKEC000000000, respectively. The type strain is BBB001^T^ (= LMG 31775 = NCTC 14418).

## Materials and Methods

### Origin of Bacteria

The bacterial screening was carried out over the rhizosphere of wild populations of *N. glauca* in Las Palmas de Gran Canaria (Islas Canarias, Spain; Coordinates UTM: 28°01′36.8″ N 15°23′19.7W) in December 2017. The soil intimately adhered to roots, and the thinner roots (diameter 1–2 mm) of four plants were pooled at random and constituted a replicate; four replicates were sampled. All materials were brought to the laboratory in plastic bags at 4°C. One gram of rhizosphere soil and thinner roots were suspended in 10 mL sterile distilled water and homogenized for 1 min in an Omni Mixer. One hundred microliters of the soil suspension was used to prepare serial 10-fold dilutions in a final volume of 1 mL; 100 μL was plated on Standard Methods Agar (Pronadisa Spain) and incubated for 4 days at 28°C. Individual colonies were selected after 36 h. To avoid duplication, isolated colonies were marked on the plate after selection. Eighty colony-forming units (cfu) were selected from each serial-dilution series, that is, from each replicate (four), constituting 320 cfu. All were isolated and grouped according to Gram staining, morphological characteristics, and sporulating capacity into four parataxonomic groups: Gram-positive endospore-forming bacilli, Gram-positive non–endospore-forming bacilli, Gram-negative bacilli, and other morphologies were grouped under “Others.” All isolates were kept at −20°C on glycerol: water (1:4) (Ramos-Solano et al., 2010). Our bacterium was isolated from these dilutions and was kept at −20°C on glycerol: water (1:4).

### Phenotypic Characterization

Our bacterium was tested for *in vitro* production of auxins, (Benizri et al., 1998), siderophores (Alexander and Zuberer, 1991), and chitinases (Rodríguez-Kábana et al., 1983; Frändberg and Schnürer, 1998) using specific culture media in which a color change reveals the biochemical trait.

To test pH and salt tolerance rates of BBB001^T^, the following procedure was followed. Growth was tested in presence of 0 to 8% (wt/vol) NaCl (at intervals of 1%) and at pH 4 to 10 (at intervals of 0.5 pH units) by supplementing TSB with appropriate buffer systems (pH 4–5.5, 0.1 M citric acid/0.1 M sodium citrate; pH 6–9, 0.1 M KH_2_PO_4_/0.1 M NaOH; pH 8.5–10, 0.1 M NaHCO_3_/0.1 M Na_2_CO_3_ (Xu et al., 2005). After autoclaving, pH medium was adjusted by the addition of NaOH/HCl 1 M before sterile filtration (pore size 0.22 μm). Analyses were carried out at 28°C for 48 h by shaking at 140 revolutions/min (rpm) in an orbital shaker and measuring OD_600_ nm every 2 h. Each well contained 200 μL of the selected medium and was inoculated at a dilution of 1:1,000 with an overnight culture of cells grown in unmodified TSB at 28°C for 24 h. Periphery pH and NaCl growth conditions previously obtained were also studied, using 5-mL tubes at 28°C for 1 week. Growth at 4 to 42°C was tested in tubes containing 7 mL TSB with shaking at 140 rpm for 8 days. All strains were precultured overnight at 28°C with TSB before suspending one loop of cells in 1 mL TSB as inoculation culture. The 7-mL test tubes were inoculated with 10 μL of the inoculation culture. The optimal growth temperature was determined using the SPECTROstar nano (BMG Labtech) and TSB under the same conditions as used for testing growth at different NaCl concentrations and pH. The range for optimal growth was determined by the length of the lag phase until the cultures achieved an increase in OD_600_ of 0.2.

The metabolic capabilities were evaluated by API 20NE and BIOLOG ECO according to manufacturer instructions. The bacterial suspension was prepared from bacterial biomass grown for 24 h in PCA resuspended in MgSO_4_ 10 mM to achieve 95% of transmittance at 620 nm. Biolog ECO plates were then inoculated with 150 μL per well. Plates were incubated at 25°C in darkness. Each well contained a substrate and tetrazolium salts, which turn violet when reduced by the activity of microorganisms. Three replicates per treatment and sampling time were performed. Kinetics of average well color development (AWCD) were used to perform a curve and to determine bacterial speed using the 31 provided substrates. From this curve, 7-day incubation time was chosen to represent the catabolic profile of the strain.

Fatty acid composition was determined according to MIDI microbial identification system (Sasser, 1990) on an Agilent 6850 gas chromatograph, provided with the MIDI microbial identification system with method TSBA6 (MIDI, 2008). This fatty acid profile was obtained from strain BBB001 grown in nutrient agar, at 28°C, for 24 h.

### Phylogenetic Analysis

To investigate the phylogenetic relationship of strain BBB001^T^ with other species of *Pseudomonas* genus and define the taxonomic allocation, three main strategies were used:

• Estimate the similarity between genomes: (a) phylogenetic analysis and delimitation of species based on the analysis of 16S rRNA and (b) ANI, AAI, hybridization DNA–DNA *in silico* or dDDH (digital DNA–DNA hybridization), difference in the content of guanine-cytosine (G + C), and frequency of tetranucleotide use.
• Phylogenetic analysis at complete genome level.
• Characterization of novel regions.

### Libraries Preparation

The DNA extraction from the lyophilized culture was performed with the REAL MicroSpin DNA Isolation kit (Durviz), strictly following the manufacturer’s instructions. A sample that did not contain a bacterial culture was processed in parallel with the other samples to verify the absence of cross contamination during DNA extraction. DNA was quantified using fluorometric methods (Qubit, Thermo Fisher Scientific).

Nextera XT Library Prep kit (Illumina) was used to prepare the library, following the manufacturer’s instructions. The fragment size distribution was then checked on an Agilent 2100 Bioanalyzer, using the Agilent DNA 1000 kit. Qubit dsDNA HS kit was used to check library concentration.

Based on these concentration data, the library was equimolarly mixed and loaded in a fraction of a MiSeq Paired-End 300 (Illumina) run.

### Quality Analysis and Sequencing Data

The FastQC 0.11.3 program was used to analyze the quality of the sequencing. Trimmomatic 0.36 (Bolger et al., 2014) was used to remove the adapters (ILLUMINACLIP option) and poor-quality regions (SLIDINGWINDOW: 5: 30). All sequences smaller than 80 base pairs were also discarded.

### *De novo* Assembly and Quality Analysis

Short sequences were assembled *de novo* using SPAdes 3.10 (Bankevich et al., 2012). SPAdes is based on the creation of graphs using the algorithm known as de Bruijin using different kmers. As recommended in the manual, kmers 21, 33, 55, 77, 99, and 127 were used; the –cov-cutoff option was set to auto and the –careful option was enabled. SPAdes use the information from the two pairs of files generated in the sequencing to transform contigs into scaffolds by introducing Ns between the contigs that can be joined.

The quality of the assemblies was analyzed using the QUAST 4.4 program (Gurevich et al., 2013).

The sequences of each sample were mapped in the assembly generated by SPAdes, to determine the coverage of each genomic region using BWA 0.7.12 (Li and Durbin, 2010). SAMtools 0.1.19 (Li et al., 2009) was used to determine the depth of sequencing and eliminate secondary or poor-quality mappings.

Qualimap 2.2.1 (Okonechnikov et al., 2015) was used to observe how the coverage is distributed throughout the genome and, in addition, to extract the specific coverage of each scaffold. To avoid possible inconsistencies or the presence of contaminating sequences, those scaffolds with less than 10 X coverage were discarded from the successive analyses. In addition, those scaffolds with a length less than 1,000 base pairs were also eliminated.

The sequencing of the complete genome on the Illumina platform MiSeq PE300 generated 986,724 sequences and a total of 290 megabases (Mb). The sequences obtained after applying the quality filters were assembled *de novo* to create larger sequences (scaffolds). Strain BBB001^T^ genome assembly generated 128 scaffolds with a total length of 5.91 Mb. The mapping of the sequences against the assembled genome confirmed that 99.94% of the sequences obtained for strain BBB001^T^ could be used for the generation of the assembly.

#### Estimates of the Degree of Similarity Between Genomes

##### Phylogenetic Analysis and Delimitation of Species Based on the Analysis of 16S

16S rRNA sequences were BLAST-queried against all strains with a validated name in the reference database at the Ezbiocloud website.

##### ANI and AAI

The first stage of this analysis consists in the comparison at the nucleotide (ANI) and amino acid (AAI) level of strain BBB001^T^ genome with all complete genomes available in the EzBiocloud database for similar genomes according to the 16S analysis previously performed. The estimates of the nucleotide identity index (ANI) were calculated using^4^. The amino acid content (AAI) was calculated at http://enve-omics.ce.gatech.edu/aai/. Values below 95% indicate a new species (Richter and Rosselló-Móra, 2009; Kim et al., 2014).

##### dDDH and Content in G + C

Based on the results of the previous section, the 26 genomes located in the fourth quartile of the distribution of the ANI values were selected (i.e., predictably, the most closely related to strain BBB001^T^), to calculate the intergenomic distances and the dDDH indexes, using the GGDC web server (Genome-to-Genome Distance Calculator). This server also calculates the difference in G + C content of genomes analyzed. Values less than 70% are indicative of significant differences (Richter and Rosselló-Móra, 2009; Kim et al., 2014).

##### Frequencies of Use of Tetranucleotides

The JSpecies software (Richter and Rosselló-Móra, 2009) was used to calculate the differences in tetranucleotide usage profiles between the genome of strain BBB001^T^ and the 26 most related genomes. Values greater than 99% are indicative of significant differences (Teeling et al., 2004; Richter and Rosselló-Móra, 2009).

#### Phylogenetic Analyses Based on Complete Genome Sequences

The genome sequence of BB001 was uploaded to the TYGS, a free bioinformatics platform available under https://tygs.dsmz.de, for a whole genome-based taxonomic analysis (Meier Kolthoff and Göker, 2019).

First, BB001 genome was compared against all type strain genomes available in the TYGS database *via* the MASH algorithm, a fast approximation of intergenomic relatedness (Ondov et al., 2016), and the 10 type strains with the smallest MASH distances chosen per user genome. This was used as a proxy to find the best 50 matching type strains (according to the bitscore) for each user genome and to subsequently calculate precise distances using the Genome BLAST Distance Phylogeny (GBDP) approach under the algorithm “coverage” and distance formula d5 (Meier-Kolthoff et al., 2013). These distances were finally used to determine the 10 closest type strain genomes for each of the user genomes.

For the phylogenomic inference, all pairwise comparisons among the set of genomes were conducted using GBDP and accurate intergenomic distances inferred under the algorithm “trimming” and distance formula d5 (Meier-Kolthoff et al., 2013). One hundred distance replicates were calculated each. Digital DDH values and confidence intervals were calculated using the recommended settings of the GGDC 2.1 (Meier-Kolthoff et al., 2013).

The resulting intergenomic distances were used to infer a balanced minimum evolution tree with branch support *via* FASTME 2.1.6.1 including SPR postprocessing (Lefort et al., 2015). Branch support was inferred from 100 pseudo-bootstrap replicates each. The trees were rooted at the midpoint (Farris, 1972) and visualized with PhyD3 (Kreft et al., 2017).

#### Characterization of *Pseudomonas* BBB001^T^ Novel Regions

The novel regions were determined using the web server Pan-seq^5^. The Novel Region Finder of this software currently identifies any genomic regions present in any of the “Selected Query” sequences (genomes of the 26 species closer in EzBiocloud) that are not present in any of the “Selected Reference” sequences (genome of BB001) and returns these regions in multi-fasta format. These comparisons are done using the nucmer program from MUMmer v3. Annotations of novel sequences were computed using eggnog-mapper (Huerta-Cepas et al., 2017) based on eggNOG 4.5 orthology data (Huerta-Cepas et al., 2018).

### Biological Assays Experimental Set Up

#### Iron Mobilization on Tomato

Tomato (*Solanum lycopersicum* L.) seeds were germinated in cocopeat with 24°C/19°C (day/night), and 15-h/9-h light–dark and kept in the greenhouse under controlled conditions throughout the 12 weeks of the experiment. Four treatments were used in this experiment: (1) control + (Fe-EDTA), (2) control− (no iron, no bacterial treatment), (3) strain BBB001^T^, and (4) the culture media free of bacteria [chelate (Q) produced by BBB001^T^]; to obtain treatments 3 and 4, strain was inoculated on nutrient broth and was grown for 24 h at 28°C under shaking, and then the culture media was centrifuged at 4,000 rpm, and the supernatant was treatment 4 (chelate), and treatment 3 was made by resuspending cells in the same volume of MgSO_4_ 10 mM solution. A total of 96 plants arranged in three replicates with eight plants each per treatment. Plants were grown under iron deficiency conditions achieved by washing substrate with bicarbonate buffer pH 8.5 to bring substrate to basic pH preventing iron absorption. After sowing, plants were watered twice a week with bicarbonate buffer pH 8.5 to maintain a high pH and with Hoagland without iron once a week. When chlorosis appeared (6 weeks after sowing), bacteria were delivered to plant roots by soil drench supplemented with iron (FeCl_3_). Three doses were delivered every 2 weeks; each dose consisted of 10 mL of strain BBB001^T^ (or chelate), at 1 × 10^8^ cfu/mL per plant supplemented with FeCl_3_ in an equivalent concentration of iron per plant to even that of Fe-EDTA. Two days after the last inoculation, plants were harvested to analyze dry weight, iron content, and photosynthetic pigments.

After 12 weeks of experiment, plants were harvested and dried, and plant weight was recorded. Leaves from plants in a replicate were pooled, and chlorophylls and carotenes were analyzed according to Lichtenthaler and Hartmut (1987), and iron was determined by atomic absorption spectrometry (method PNT.08.01).

#### *Olea europaea* Production in Intensive Orchards Under Water Limitation

A 12-year-old intensive orchard of *O. europaea* var. Arbequina in Toledo (Spain) (Coordinates UTM 393782–4343510) was used for this experiment. Two lines of 100 m each were selected, and treatments were marked within the two lines on a random block design. Treatments consisted in three replicates of seven trees; BBB001^T^ treatment was root inoculated (500 mL per tree with a bacterial concentration 1 × 10^8^ cfu/mL) every 2 weeks from April to October, and controls were mock inoculated with water. Regular watering consists of four times a week, each 2 L/h for 3 h; in experimental trees, one time was skipped to decrease water input by 25%. Olives were harvested at the end of October, collecting all olives from the seven trees in the replicate, which were weighed, and fat content determined with Soxhlet. Production (kg/ha) was estimated calculating production per plant and considering orchard’s plant density (1,600/ha).

### Statistical Analysis

To evaluate treatment effects on all variables, one-way analysis of variance was performed. When significant differences appeared (*p* < 0.05), LSD test (least significant difference) Fisher was used. Statgraphics Plus for Windows was the program used.

## Data Availability Statement

The datasets presented in this study can be found in online repositories. The names of the repository/repositories and accession number(s) can be found below: https://www.ncbi.nlm.nih.gov/bioproject/PRJNA705568.

## Author Contributions

FJGM conceptualized the manuscript. EG-A and IH carried on the bacterial isolation. EG-A analyzed the genomic data. JL, AG-V, and EG-A developed tomato bioassay and drafted the manuscript. EG-A, BR-S, and FJGM developed olive assay. FJGM and BR-S wrote and supervised the final version. All authors contributed to the article and approved the submitted version.

## Conflict of Interest

The authors declare that the research was conducted in the absence of any commercial or financial relationships that could be construed as a potential conflict of interest.

## Publisher’s Note

All claims expressed in this article are solely those of the authors and do not necessarily represent those of their affiliated organizations, or those of the publisher, the editors and the reviewers. Any product that may be evaluated in this article, or claim that may be made by its manufacturer, is not guaranteed or endorsed by the publisher.

## Footnotes

1
https://www.ncbi.nlm.nih.gov/bioproject/PRJNA705568

2
https://antismash.secondarymetabolites.org/#!/start

3
https://lpsn.dsmz.de/species/pseudomonas-qingdaonensis

4
https://www.ezbiocloud.net/tools/ani

5
https://lfz.corefacility.ca/panseq/page/index.html
