# Supplementary material for: Pseudomonas palmensis sp. nov., a Novel Bacterium Isolated From Nicotiana glauca Microbiome: Draft Genome Analysis and Biological Potential for Agriculture
Source: Front Microbiol. 2021 Aug 20;12:672751. doi: 10.3389/fmicb.2021.672751 (PMC8417607; doi:10.3389/fmicb.2021.672751)
Supplement: Supplementary file 1 [file Data_Sheet_1.PDF]

Supplementary material

Table 1. Bacterial Growth under different pH conditions and salt concentration after 24h of incubation

pH	Growth (cfu/ml)	% salt (w/v)	Growth (cfu/ml)
2	0	2	1×10^9
4	0	4	1×10^9
6	2×10^9	6	1×10^6
8	4×10^9	8	0
10	3×10^9	-	-
12	0	-	-

Table. 2.Phenotypic characteristics measured using Biolog™ ECO plates. + indicates positive use of this carbon source; - indicates a negative use of this carbon source, at 24 h of incubation.

pyruvatic acid methyl ester	+
Tween 40	+
Tween 80	+
α-cyclodextrin	-
glycogen	+
D-cellobiose	+
α-D-Lactose	+
α-Methyl-D-Glucoside	+
D-Xylose	+
i-Erythritol	-
D-Mannitol	+
N-Acetyl-D-Glucosamine	+
D-glucosaminic acid	+
Glucose-1-Phosphate	+
D.L-α-glycerol phosphate	+
D-galactonic acid γ-lactone	-
D-galacturonic acid	+
2-hydroxybenzoic acid	-
4-hydroxybenzoic acid	+
γ-hydroxybutyric acid	+
itaconic acid	-
α-ketobutyric acid	-
D-malic acid	+
L-arginine	-
L-asparagine	+
L-phenylalanine	+
L-serine	+
L-threonine	-
glycyl-L-glutamic acid	+
phenyl ethylamine	+
putrescine	+
